# Integrated Pest Management (IPM) for Small-Scale Farms in Developed Economies: Challenges and Opportunities

**DOI:** 10.3390/insects10060179

**Published:** 2019-06-21

**Authors:** Tessa R. Grasswitz

**Affiliations:** Sierra Biological, 2825 Swett Road, Lyndonville, NY 14098, USA; tess@sierrabiological.com

**Keywords:** small farms, non-chemical pest management, urban agriculture, organic farming, exotic pest detection

## Abstract

Small-scale farms are an important component of agricultural production even in developed economies, and have an acknowledged role in providing other biological and societal benefits, including the conservation of agricultural biodiversity and enhancement of local food security. Despite this, the small-farm sector is currently underserved in relation to the development and implementation of scale-appropriate Integrated Pest Management (IPM) practices that could help increase such benefits. This review details some of the characteristics of the small farm sectors in developed economies (with an emphasis on the USA and Europe), and identifies some of the characteristics of small farms and their operators that may favor the implementation of IPM. Some of the challenges and opportunities associated with increasing the uptake of IPM in the small-farm sector are discussed. For example, while some IPM tactics are equally applicable to virtually any scale of production, there are others that may be easier (or more cost-effective) to implement on a smaller scale. Conversely, there are approaches that have not been widely applied in small-scale production, but which nevertheless have potential for use in this sector. Examples of such tactics are discussed. Knowledge gaps and opportunities for increasing IPM outreach to small-scale producers are also identified.

## 1. Introduction

Sixty years after the concept of ‘integrated control’ was first introduced by Stern et al. [1], the field of ‘integrated pest management’ (IPM) has expanded beyond the original vision of those authors to embrace not only chemical and biological control, but a wide variety of additional approaches to pest suppression. However, in most developed economies, IPM-related research and extension programs are now heavily focused on large-scale agriculture, with corresponding efforts for small-scale farmers being conducted primarily in less-developed nations.

Consequently, small-scale producers in developed economies are now relatively poorly served with regard to their IPM-related needs, despite the large number of farms that they represent. A recent survey in the European Union, for example, showed that two-thirds of all farms were less than 5 hectares in size [2], while, in the US, slightly more than half of all farms are reported to be ‘very small’, with annual farm sales of under $10,000 [3]. Collectively, such farms make a significant contribution to food production and local food security: on a global basis, it is estimated that approximately 30% of the world’s food supply is produced on farms less than 2 ha in size [4]. Small farms also contribute to other biological and societal benefits, including the conservation of agricultural biodiversity and the maintenance of culturally important crops and landscapes. A greater investment in the development and implementation of scale-appropriate IPM practices for such farms could increase such benefits.

This review therefore addresses the perceived challenges and opportunities regarding the development and adoption of IPM practices on small-scale farms in developed economies, with a primary focus on the United States and Europe. A brief overview of the small-farm sectors in these areas, together with some of their principal characteristics, is provided for contextual purposes, since there is consensus in the literature that small farms differ in fundamental ways from larger-scale agri-businesses, and these differences may affect the adoption of various components of IPM. Discussion of individual IPM tactics is limited to those that may be easier (or more cost-effective) to implement on a small scale, or which have not historically been widely used in small-scale production, but which nevertheless have potential for use in this sector. Pest management tactics that are considered equally applicable to large or small-scale production are omitted.

## 2. The Small Farm Sector in Developed Economies

There is no universal definition of a ‘small farm’ [5,6]. Most potential defining characteristics are to some extent context dependent—for example, what may be considered a ‘small’ farm may differ according to geographic location (country or region), or the nature of the farm enterprise (e.g., extensive livestock production compared to intensive vegetable production).

Hence, although data on ‘farm size’ are collected in most developed countries for administrative or policy-related purposes, the basis on which ‘size’ is defined is not limited to agricultural land area *per se*, but may also include the amount and/or nature of labor or ownership (e.g., family versus non-family), the economic scale (output) of the farm, and/or its degree of market involvement [5]. In the USA, for example, the US Department of Agriculture (USDA) defines as a ‘farm’ any area ‘from which $1000 or more of agricultural products were produced and sold, or normally would have been sold, during the year’ [7]. This basic definition is expanded to encompass a total of seven farm types through a combination of three metrics: gross cash income, the primary occupation of the principal operator, and whether or not the farm is family owned and operated [7]. Under these criteria, ‘small’ farms are considered to be those generating less than $350,000 in annual gross cash farm income, although a ‘low sales’ category (generating annual gross cash farm income of less than $150,000) is recognized within ‘farms whose operator’s primary occupation is farming’ [8]. Slightly more than half of all US farms are reported to be ‘very small’, with annual farm sales under $10,000 [3]. Note that the actual area of agricultural land is not considered in these farm classifications, although the 2012 US agricultural census revealed that 11% of all US farms were approx. 3.6 ha (nine acres) or less in size [9].

Elsewhere, small farms have been defined by various authors and entities as those with an agricultural land area of 2 or less ha (approx. 5 acres) or, in some cases, up to 10 ha [10,11,12,13]. This multiplicity of definitions complicates comparisons between countries and regions in relation to issues concerning ‘small farms’, but, for the purposes of this review, the focus is on farms of approximately this size.

Despite the inherent difficulties associated with defining small farms, there is general agreement in the literature that the small farm sector is characterized by considerable diversity (e.g., in farmer demographics, farm enterprises, and production methods) and differs in fundamental ways from larger-scale agri-businesses (which generally have better access to infrastructure, capital, and information) [6,12]. D’Souza and Ikerdey [14] list three characteristics other than size that commonly distinguish small farms from their larger counterparts, namely: (i) the ‘intensity of the grower-nature relationship’, (b) the diversity of plant and animal life, and (c) the diversity of income sources on small farms relative to larger ones.

In relation to farmer demographics, the 2012 farm census conducted by the US Department of Agriculture found that higher proportions of women, Hispanic, Native American and black farmers are associated with small acreage or low-sales farms compared to the overall national averages for these farm categories. For instance, 54% of woman-owned farms were less than approximately 20 ha (50 acres) in size (the smallest size class given), compared to 39% of all farms in the census as a whole; similarly, 76% of women-owned farms generated annual farm sales of less than $10,000 per year, compared to 56% of farms in the census as a whole [15]. For Hispanic farmers, these metrics were, respectively, 58% and 68% [16], for American Indian farmers, 57% and 78% [17], and for black farmers, 49% and 79% [18]. In addition, while 11% of all US farms were between 0.4–3.6 ha (1–9 acres) in size, for farms reporting that more than 50% of their total sales were organic, 18% were in this size class [19]. A high level of interest in organic growing amongst small-scale US farmers was also found in two recent surveys [20,21].

In the European Union (EU), data from the most recently analyzed farm structure survey indicated that smaller farms (defined in economic terms) tended to include a greater diversity of farm enterprises (mixed cropping, mixed livestock, or mixed crops and livestock) compared to larger farms [22]. In both the USA and EU, many small-scale farmers derive part of their income from off-farm employment [8,22], and, in both regions, small-scale growers include some refugees or members of other immigrant communities who may face language and other barriers [23]

An additional layer of complexity in the small farm sector lies in location, as such farms are found not only in ‘traditional’ (rural) farming areas, but also in urban or peri-urban areas (the latter being defined as a transitional zone characterized by lower population densities and poorer infrastructure compared to cities, but with a lower proportion of agricultural or undeveloped land compared to rural areas [24]). Farms in these three zones have different characteristics and their operators face somewhat different challenges and opportunities [24]. Urban farmers in particular may be motivated by social or environmental concerns as much as (or more than) potential profit.

As discussed below, the high level of heterogeneity in the characteristics of owner-operators of small farms, and the diversity of enterprises that they embrace, represent both challenges and opportunities for increasing the adoption of IPM in this sector.

## 3. Challenges in Increasing the Adoption of IPM on Small Farms

### 3.1. Lack of Knowledge and Appropriate Research/Technical Support

A lack of fundamental horticultural knowledge is most commonly reported in relation to small-scale urban farmers, who may be more likely than their rural counterparts to come from non-farming backgrounds [25], and who may be disadvantaged in other respects (e.g., through language barriers). Basic agricultural training for these growers is often provided by non-profit organizations, sometimes in partnership with municipal authorities or other service providers (e.g., agricultural extension services) [24]. However, such training usually includes only very basic information on pest management, and managing pests and weeds are often among the most important challenges reported by these growers [20,25,26,27]. Orpitz et al. [24] go as far as to express concern that inexperienced or short-term practitioners of urban agriculture may lack the knowledge needed for the responsible use of pesticides and fertilizers.

Small-scale farmers in more rural areas also tend to ‘fall through the cracks’ in relation to assistance with IPM implementation. In the USA, for example, although most state land grant universities generally have both small-farm and IPM programs, most of the former include very little IPM programming, while state IPM research and extension efforts are often heavily focused on large-scale agriculture, with little overt attention paid to the needs of small-scale growers.

For advisory information to be useful, it must be both accessible and relevant to the target audience. If significant barriers exist (including, for example, language, work schedules or culturally distinct learning styles), various approaches may be needed to make advisory information accessible to the target audience(s). For example, when classroom-based workshops failed to attract Hmong refugee farmers in Washington State (USA), Extension educators instead taught Hmong youth how to produce educational videos for the growers. The videos were considered more convenient and culturally appropriate, as, in Hmong culture, the written language is seldom used, and farmers traditionally learn primarily through storytelling and visual means [23]. However, where resources are limited, the necessary funding and personnel (with appropriate linguistic and cultural backgrounds) may not be available to devise and implement extension programs tailored to the needs of individual demographic groups.

In a similar way, for advisory information to be considered ‘relevant’ it must be ‘likely to provide a solution to the specific problem faced by the farmer…a solution that suits [their] objectives and interests’ [28]. ‘Generalized’ recommendations that fail to consider farmer constraints are unlikely to be adopted. In IPM—as in other areas of production—the applicability of certain strategies or tactics is scale-dependent, and what is relevant to large-scale production is not always relevant to, or appropriate for, small-scale production (and vice versa).

That said, in the US, the current outlook for improving technical support for IPM on small farms looks somewhat brighter for the urban than the rural sector, largely because the perceived wider benefits of urban agriculture have resulted in increased political support—and hence funding—for this sector [25,27,29].

In Europe, the trend towards the gradual commercialization and ultimate privatization of farm advisory services, which began in the 1980s, has been disproportionately detrimental to small-scale farmers, most of whom cannot afford to pay for advice that was formerly provided free of charge [6,28]. Even where advisory services are available and affordable, some small-scale growers are hesitant to use them because of a perception that such services are primarily intended for large-scale growers [28]. Often, too, the advice provided by such agencies is focused mainly on accessing farm subsidies or meeting EU regulatory requirements, rather than on production aspects of farming [6]. Furthermore, there is often no effective means by which the information needs of small-scale producers can be effectively conveyed to policy makers [28].

Lack of access to information provided via the internet, or lack of experience/familiarity with such information sources, can also be a barrier, particularly in rural areas where internet services may be absent or expensive, even in developed economies [28]. Furthermore, for some groups of small-scale farmers, computer- or telephone-based information delivery may actually be culturally unacceptable [30]. Even where internet access is readily available, however, it may still take growers a considerable time to compile, evaluate and authenticate the information needed to address a new problem [25]. This can be a significant drawback in relation to pest and disease management, where timely interventions are often necessary. Time-sensitive information of this nature could usefully be disseminated on a local scale through regular farmer meetings, social networks, or field days, and organizing such events is perhaps one of the most useful ways in which formal farm advisors could assist small-scale growers [6]. Indeed, numerous studies of farmer information sources have indicated that ‘other farmers’ are often one of their most important resources [30,31] and scheduling regular meetings could help facilitate this exchange. While some farmers may not be willing to share their knowledge, others may bring innovative approaches or expertise from previous training, or off-farm employment [6]. Regular group activities of this kind could also help overcome other barriers, such as farmer isolation, language difficulties, and scheduling (e.g., for producers with off-farm employment).

Lack of IPM research relevant to small farms is perhaps a more serious problem than the provision of advisory services. In the US, federal grant programs that support IPM-related research emphasize large-scale, multi-state collaborations on shared issues with large economic impacts. While in some cases, practices developed for large-scale producers can subsequently be adapted for use by smaller-scale producers with little modification, in other cases, the best practices for the latter may be completely different [32]. Furthermore, where scale-appropriate modifications of research results are required, additional research (with the need for associated funding and expertise) will inevitably result in additional delays in implementation at the farm level.

In a recent survey of extension personnel in California, a lack of current research-based educational materials was identified by nearly a quarter of all respondents as being a significant barrier to assisting small-scale urban farmers [25]. In Europe, the *de facto* transfer of farm advisory services to independent consultants, input suppliers and similar entities, has resulted in a lack of local research relevant to small farms and has also reduced feedback from producers to applied researchers in academic institutions [28]. The latter aspect is important, since developing solutions that are both relevant to small farms and consistent with the farmers’ objectives requires direct interaction between the farmers and the researchers and advisors who seek to assist them. The increasingly commercialized nature of public/academic research militates against research specifically targeted at the needs of small-scale producers [33].

On-farm participatory research (perhaps based on a modified ‘Farmer Field School’ approach [34,35]) can be one way of developing locally relevant IPM approaches for small-scale farmers [36]. The US Department of Agriculture provides support for such an approach via its Sustainable Agriculture Research and Education (SARE) Program, and similar activities occur in Europe (e.g., via the United Kingdom’s ‘Innovative Farmers’ network and others supported by the European Innovation Partnership for Agricultural Productivity and Sustainability (EIP-AGRI)). Such programs can help leverage relatively small amounts of funding, although the labor and time needed to conduct the research can still be a barrier for many small-scale growers.

### 3.2. Availability of Scale-Appropriate Pest Management Inputs

The ability of small-scale farmers to implement some pest management tactics may be limited not only by cost, but also by the availability of certain inputs in scale-appropriate pack sizes. For example, although research has demonstrated the efficacy of pheromone-based mating disruption for management of peachtree borer (*Synanthedon exitiosa* (Say) (Lepidoptera: Sesiidae)) even in very small orchards (0.08 ha) [37], at present the pheromone dispensers are only available in pack sizes that far exceed the needs of growers with orchards of this size. Similarly, some insecticides approved for organic production require pH buffering for use in alkaline water; however, in some countries, organically-approved buffering agents are available only in very large containers (again, containing far more than would be needed by a small-scale grower), with limited availability and very high shipping costs. The dual issues of inappropriate pack sizes and limited distribution networks can be serious impediments for small-scale producers seeking specialized pest management products. By the same token, however, these same issues could be viewed as opportunities for those input manufacturers or distributors willing and able to address them.

A related problem is that small-scale producers lack the advantages that large-scale farmers often have in negotiating with suppliers of agricultural inputs (e.g., for discounts on bulk purchases). This might be overcome if individual producers were to form co-operatives or grower associations to increase their bargaining power [32].

### 3.3. Lack of Visibility and Political ‘Voice’

To a large extent, both of the preceding challenges stem from the same root cause: in developed countries in particular, small-scale farmers individually lack the visibility and political influence of their larger-scale counterparts. While the expansion of organic production and urban agriculture have to some extent helped to re-dress this balance, the basic problem remains. As suggested above, the formation of co-operatives or grower associations by groups of small-scale producers might enhance their ability to better represent their needs to legislative bodies and to research and educational institutions in regards to agricultural policies and IPM priority-setting.

## 4. Opportunities: Factors Favoring Adoption and Development of IPM on Small Farms

### 4.1. On-Farm Biodiversity

In ecological theory, the ‘diversity–stability’ hypothesis predicts that more diverse ecological communities will be more stable than less diverse communities [38]. Over the past 50 years, this—and related—hypotheses have stimulated considerable associated research, both in ecological contexts and, to a lesser extent, in agricultural systems [39,40].

Research on the effects of plant diversity in relation to pest management has encompassed not only the effects of multi-species cropping systems *per se*, but also the effects of enhancing non-crop farm habitat to support beneficial species. In many cases, there is evidence to support the beneficial effects of plant diversity on pest populations [40], but there are exceptions [41], and it is important to consider such effects in the context of specific pests and farm systems [42,43].

In relation to small farms, a recent meta-analysis indicated that small-scale farmers tend to grow a high diversity of crop species, with the highest diversity (globally) being found on farms of 1–2 ha in size [4]. Small, highly diversified farms readily lend themselves to potential IPM approaches such as intercropping, under-sowing, or cultivar mixtures [44], which can be effective components of pest management, but which are often difficult to implement on a large scale. Judicious use of cover crops/living mulches and adjusting cropping plans to ensure that closely related crops are separated either spatially or temporally would allow small-scale growers to capitalize on the disruptive effects of non-host plants on crop colonization by more specialized crop pests [45,46].

Practices intended to enhance the diversity of non-cropped areas of the farm are similarly adaptable to small acreages. Small-scale farmers often have a strong stewardship ethic, and may be willing to forgo some level of profit to implement such measures. In some cases (e.g., creating shelterbelts or hedgerows), potential costs could be offset by incorporating novel or under-utilized trees or shrubs that provide both floral resources for beneficial insects and edible fruits for potential sale. Depending on location, such species might include, for example, jujube (*Ziziphus jujuba* Mill.) (Rhamnaceae), Juneberry (*Amelanchier alnifolia* Nutt.) (Rosaceae) or Elderberry (*Sambucus* spp.) (Adoxaceae). Efforts to develop local or regional planting lists that include such plants are likely to be well-received by small-scale growers.

Financial incentives may also help to increase adoption of diversity-enhancing practices for non-cropped areas of small-scale farms [47]. In both Europe and the US, government-supported ‘agri-environment’ schemes (such as the UK’s Countryside Stewardship Grants and the USDA’s Environmental Quality Incentives Program (EQIP)) provide cost-share payments for several practices of this kind (e.g., creation of hedgerows, shelterbelts, ‘beetle banks’, and strips of flowering ‘insectary’ plants to support native natural enemies). Some small-scale farmers are highly motivated to diversify the non-crop areas of their farms. A recent study in California, for example, found that deliberate biological enhancement of field-edge habitats was significantly more likely to occur on smaller versus larger farms, and to be implemented more by organic farmers than by conventional producers [48]. Similarly, small-scale farmers in Missouri listed creating habitats for beneficial insects and using biological control for insect pests as their top two IPM topics of interest [20].

There are thus multiple ways in which plant diversity (crop or non-crop) can be manipulated to contribute to pest management. Since these methods have been the subject of several recent reviews [39,43,49,50,51,52], they are not discussed in detail here. However, in addition to the measures listed above, in some cases, trap-cropping may also be appropriate for small-scale farms. This technique seeks to prevent or reduce damage to the target crop by attracting one or more key pests to a limited area of a more attractive host plant (species or cultivar), or, in some cases, an earlier planting of the main crop. Once concentrated on the trap crop, a suitable control technique is typically applied to this limited area to suppress the pest, thereby potentially reducing the need for additional control measures on the main crop [53]. One drawback of this technique is that it may require as much as 10–15% of the crop area to be planted with the trap crop [43], which would be impractical for many small-scale growers. However, the trap crop area can be reduced if the plant used is particularly attractive to the target pest, or if its attractiveness can be increased by deploying attractant lures. For example, significant reductions of cucumber beetles (*Acalymma vittatum* (F.), and *Diabrotica undecimpunctata howardii* Barber) (Coleoptera: Chrysomelidae) were achieved even when trap crops represented less than 1% of the crop area by applying feeding attractants to plants of highly attractive trap crop cultivars that had also been treated with systemic insecticides [54]. In general, however, there are few examples of successful trap crop systems at this scale [53].

However, the high crop diversity found on many small-scale farms may favor adoption of IPM in another way: since both multi-species cropping systems and IPM are widely viewed as being knowledge-intensive and more difficult to manage than simplified systems [35,55,56], growers who are comfortable with managing complex growing systems might be expected to be more receptive to the complexities of implementing IPM. While this hypothesis does not seem to have been widely tested, it is supported by studies of vegetable growers in Florida, Michigan and Texas, in which a positive relationship was found between the number of crops grown and the likelihood of adopting IPM [57]. The pre-disposition of many small-scale growers to use organic farming methods (with or without formal certification) [20,21] would also tend to favor adoption of many of the non-chemical tactics emphasized in IPM.

In addition, many small-scale farmers help maintain crop genetic biodiversity through their seed-saving activities, which may include selecting for resistance to pests, diseases and abiotic stressors [58,59]. This role of small-scale growers (particularly in relation to the conservation of traditional (‘heirloom’) varieties or locally-adapted land races) is well-documented in developing countries [59,60], but has received less attention in post-industrial economies. However, even in developed countries, many small-scale producers save their own seed, and some immigrant farmers maintain more unusual crops for which local seeds may be hard to find or expensive [61,62]; the latter are therefore also being selected for locally adapted traits that may include resistance to arthropod pests and/or pathogens. In relation to these latter traits, seed saving by small-scale organic producers may be particularly important, since their crops are grown with minimal exposure to pesticides and other synthetic inputs.

Finally, the high crop diversity found on many small farms—particularly in urban or peri-urban environments—presents a little-appreciated opportunity for the early detection of new exotic pests and diseases. For example, from 2010 to 2014, members of US Western Small-Farm IPM Working Group conducted various small-farm IPM pilot projects that resulted in the detection of several pests and diseases that had not previously been recorded in the participating states. In New Mexico, for example, the first state records for spotted wing drosophila (*Drosophila suzukii* Matsumura) (Diptera: Drosophilidae) and the cereal aphid *Sipha maydis* Passerini (Hemiptera: Aphididae) were both obtained on small-scale, diversified urban farms participating in the New Mexico IPM pilot project; similarly, various new vegetable diseases were detected on similar farms by working group members in Utah, and a new viral disease of dragon fruit (*Hylocereus* spp.) (Cactaceae) was found in California [63]. There are three factors that make small urban- or peri-urban farms conducive to hosting to new invasive species: (1) such farms are often situated close to major transportation hubs and trade routes by which new exotic pests may enter individual states or regions; (2) they are often farmed with minimal use of pesticides, either because of grower preferences for organic methods, or because of a lack of registered products, and (3) many urban growers plant exotic crops (e.g., for local ethnic markets or restaurants), which can include preferred hosts for exotic pests. Making use of sites of this kind for programs such as the USDA-APHIS Cooperative Agricultural Pest Survey (CAPS) might result in higher levels of interception of target species than surveillance conducted on larger-scale commercial farms that may be more remote from major transportation routes, grow fewer crops, and/or use higher levels of pesticides.

### 4.2. Pest Detection and Response Times

Regular scouting for pests and diseases is an important component of IPM [64] and, theoretically at least, potential problems may be detected in a more timely manner on small farms where, it has been argued, growers have more time to devote to, and interact with, individual crops [14]. While there is some evidence indicating that small-scale farmers often do scout for pest problems (either formally or informally) [20], the effectiveness of these practices may be limited by their pest recognition skills, with plant diseases in particular often being difficult for growers to diagnose [20]. Targeted programs by Extension workers or other advisors could have considerable impact in this regard.

On a similar basis, it can be hypothesized that small-scale growers should be able to make more timely interventions for pest management than can large-scale growers, who may be constrained by the availability of labor and/or spray equipment if they have large acreages to cover in a limited time (for example, for the preventative application of fungicides for plant pathogens). On the other hand, the reverse may be true for the many small-scale farmers who have some form of off-farm employment. These might be fruitful questions for future research on IPM implementation on small farms.

### 4.3. Scale-Appropriate IPM Tactics

Some ‘traditional’ IPM tactics (such as pest-resistant crop cultivars) may be equally applicable and easy to implement in virtually any scale of production. However, there are others (such as intercropping and under-sowing) that may be easier and/or more cost-effective to implement on a smaller scale.

Furthermore, either by choice or necessity, many small-scale farmers are highly motivated to minimize their use of pesticides, for reasons that may include (amongst others) a preference for/commitment to organic methods, a lack of access to suitable pesticides in appropriate pack sizes, and/or possible restrictions on pesticide use in urban settings.

Such considerations present considerable opportunities for the creative use of existing non-chemical pest management tactics, or for the development of new ones. The following discussion highlights those IPM tactics that (i) may be easier to implement in (or which may be particularly appropriate for) small-scale production systems, or that (ii) have not previously been widely adopted in small-scale agriculture, but which nevertheless have some potential for use in this sector. IPM tactics considered to be equally applicable for use on both small- and large-scale farms have been omitted.

#### 4.3.1. Pest Exclusion

Physical barriers that prevent insect pests from reaching host crops are becoming more widely used by growers of specialty crops—particularly for fruit crops, but also to some extent for vegetables as well.

Physical exclusion can be a very cost-effective and successful component of IPM for small-scale producers, as the time and effort expended in installation can be offset by reducing or eliminating the need for further interventions for the target pest(s) for much, if not all, of the growing season. The fact that many physical barriers can be re-used for multiple years also helps offset the initial cost of the materials.

Nevertheless, certain precautions must be taken if exclusion techniques are to be successful, and in general these make such approaches more appropriate for small-scale farms than for larger ones. For example, it is necessary to ensure that nets or other covers are in place well before the anticipated date of crop colonization by the target pest(s), while also trying to ensure that few or no other pests or pathogens are present on the crop at the time of covering [65]. The latter is particularly important in the case of potential virus vectors, although control of other pests already present on the crop may subsequently be impaired by the unintended exclusion of natural enemies once the cover is in place [66,67,68].

In addition, (with a few exceptions, as discussed below), care must be taken during installation to ensure that the crop is fully covered, and, where appropriate, that there is sufficient separation between the cover (e.g., netting) and the crop to prevent pests ovipositing through the mesh [65,69]. During the growing season, covers should also be checked regularly for holes or tears (which should be promptly repaired), and, where necessary, to ensure that an effective seal is maintained where the barrier meets the soil.

With these caveats in mind, various approaches to pest exclusion have been developed that are particularly appropriate for small-farms, as discussed below.

##### Crop Covers

So-called ‘floating’ row covers of non-woven/spun polypropylene (also known as ‘horticultural fleece’) have been in widespread use in agriculture since the 1990s [70], mainly for frost protection and other horticultural benefits, but also for pest exclusion. They are particularly useful for early season protection against seedling pests, when they can be installed shortly after sowing and provide additional benefits such as faster germination (as a result of increased temperature) and reduction of surface soil compaction following heavy rain [71]. When used in this way to protect newly sown carrot seedlings from carrot weevil (*Listronotus oregonensis* (Le Conte) (Coleoptera: Curculionidae), approx. 40 days under cover was sufficient to reduce carrot weevil damage by 65–75%. With this level of control, the authors considered that no insecticides would be needed under low to medium pest pressure [71]. Similarly, covering newly seeded plots of radish (*Raphanus sativus* L.) (Brassicaceae) with floating row covers completely excluded cabbage root fly (*Delia radicum* L.) (Diptera: Anthomyiidae) and reduced flea beetle (*Phyllotreta* spp.) (Coleoptera: Chrysomelidae) damage by 60% compared to uncovered control plots [72]. In situations such as these, early installation of a floating row cover could save small-scale growers both time and money if later insecticide applications could be avoided.

One disadvantage of floating row covers is that, later in the season, the heavier fabrics (which are more resistant to accidental tearing) may subject the crop to unacceptably high temperatures, with negative effects on plant health and yield [72]. In some cases, substituting a lighter cover might still provide acceptable exclusion, but the fabric may not be sufficiently robust for use over more than one season. Another option might be to use small metal hoops to support the fabric (forming a low tunnel), or to cover such hoops with fine-mesh insect netting rather than a non-woven fabric [73].

Pest exclusion with nets of various kinds has been the focus of considerable research in recent years, and several approaches are suitable for small-scale production. In berry crops, for example, netting is being used in various parts of the world to exclude ovipositing adults of spotted wing drosophila (SWD) from ripening fruits of susceptible hosts, which include blueberries, blackberries, raspberries, cherries, and elderberries. Exclusion netting for SWD management has been shown to be very effective in both experimental and small-scale commercial plantings of blueberries [74,75,76,77], while in raspberries, it delayed crop colonization for approximately 3 weeks—long enough to potentially save growers several applications of insecticide [78]. The initial investment required for netting such crops can be quite high, but the fact that the nets can be re-used for multiple years helps offset the costs over time [76,77,78].

Witkowska et al. [65] discussed other benefits accruing to vegetable producers when radishes were grown under nets to exclude cabbage root fly and flea beetles (*Phyllotreta* spp.); these included improved protection against frost and vertebrate damage, improved soil and water conditions, savings on labor to sort the crop prior to packing (because of lower damage), and beneficial changes in crop density that were initially adopted to increase cover efficiency. The latter innovation resulted in a doubling of yield while using 33% less land [65].

In tree fruit systems, research on the potential of netting for pest management (either on individual rows or over entire orchard blocks) has also been stimulated—at least in part—by the fact that various horticultural benefits of the technique have already been demonstrated. The latter include manipulation of tree growth, yield and crop quality [67] as well as protection from weather-related damage (e.g., by hail, wind or sunburn) [67,79,80,81]. The value of such benefits to growers may favor the adoption of nets as pest management tools by offsetting the initial costs, particularly as some orchard nets may last for as long as 10–15 years [66,82]. Nets may also be more cost-effective where exotic invasive species (such as spotted wing drosophila or brown marmorated stink bug (*Halyomorpha halys* (Stål) (Hemiptera: Pentatomidae) cause heavy losses and incur high management costs [82].There is thus increasing interest in, and adoption of, exclusion nets for pest management [69,81,83] and this in turn may further reduce unit costs. Nevertheless, results in tree fruit have been somewhat variable [66]. Alaphilippe et al. [81], for example, reported that nets gave good control of codling moth (*Cydia pomonella* (L.) (Lepidoptera: Tortricidae) in both apples and pears, but resulted in increased populations of some indirect pests, including rosy apple aphids (*Dysaphis plantaginea* (Passerini)) (Hemiptera: Aphididae), woolly apple aphids (*Eriosoma lanigerum* Hausmann) (Hemiptera: Aphididae), and summer fruit tortrix *Adoxophyes orana* (Fischer von Röslerstamm) (Lepidoptera: Tortricidae). In the case of the two aphid species, this may in part have been due to exclusion of natural enemies [66,67], although changes in microclimate under the netting may also have had an influence; a similar result was observed when fine mesh exclusion netting was used in high tunnels [68]. Exclusion netting has also been used on an experimental basis for managing various tephritid fruit flies attacking tree fruits, including European cherry fruit fly (*Rhagoletis cerasi* (L.) (Diptera: Tephritidae) in Europe [84] and Queensland fruit fly (*Bactrocera tryoni* (Froggatt)) (Diptera: Tephritidae) in Australia [85].

##### Photoselective Netting

Colored (‘photoselective’) shade nets may offer another useful tactic appropriate for small-farm IPM in greenhouses or high tunnels. These nets were developed mainly to manipulate crop growth by modifying the characteristics of the light reaching the plants grown beneath them [86,87]. While they do not physically exclude insects, some of the net colors can affect the propensity of pests to colonize certain crops. For example, in a series of experiments conducted over several years, levels of aphids (*Myzus persicae* (Sulzer) and *Aphis gossypii* Glover (Hemiptera: Aphididae), and whiteflies (*Bemisia tabaci* (Gennadius) (Hemiptera: Aleyrodidae), were consistently 2–3 times lower on pepper and tomato crops grown under yellow or ‘pearl’ colored nets than on those grown under black or red nets of similar shading capacity. Similarly, levels of several viral diseases transmitted by these insects were reduced by 2–10 times in crops grown under the yellow or pearl nets compared to those grown under black or red nets [88].

Photoselective nets have also been used experimentally to manage codling moths in apple orchards, where they were also found to affect the abundance and species composition of orchard carabid communities: the highest total number of ground beetles was captured under yellow nets [89], but spider communities were not affected [90]. These results suggest that it may be possible to use photoselective nets to manipulate predator–prey ratios in favor of biological control, either in open field situations or in greenhouses. This subject is worthy of further investigation.

##### Barrier Fences

For insects that migrate into the crop by flying low to the ground, it may be possible to avoid some of the disadvantages of completely enclosing a crop in netting by erecting low ‘barrier fences’ around the perimeter of the field. For example, in experimental trials, significant reductions in damage to radish by cabbage root fly (*D. radicum*) were achieved by surrounding the crop with low (135 cm high) fences made of nylon window screen, with the top edges of the fence enhanced on both sides by 25 cm-long mesh overhangs supported at 45° angles to the top [91]. These overhangs helped prevent flies that were deflected by the fence from clearing its upper edge as they moved upwards. The level of suppression in this case was not sufficient for the fencing alone to serve as a stand-alone tactic, but it nevertheless shows promise as one component of an IPM program for cabbage root fly and similar pests [92]. This technique has also been used experimentally to protect broccoli (*Brassica oleracea* L. var. *italica*) and kohlrabi (*Brassica oleracea* var. *gongylodes*) (Brassicaceae) from the swede midge (*Contarinia nasturtii* (Kieffer)) (Diptera: Cecidomyiidae)), providing significantly better control than three applications of spinosad [93]. Low barriers such as these should also permit free movement of most beneficial insects, although they may impede crop colonization by some flightless Carabid species [94].

##### Insecticidal Nets

Long-lasting insecticide-treated nets (LLITNs) offer another variation on the exclusion technique, which may also prove a useful addition to the IPM toolbox for small-scale farms. These nets contain an insecticide (usually a pyrethroid) that is incorporated into the fiber (polyethylene or polyester) prior to the manufacture of the netting; the insecticidal activity can persist for at least one field season [95], and possibly longer under some circumstances.

Experimentally, use of such nets in high tunnels was found to reduce the incidence of two aphid-transmitted virus diseases of cucurbits, while being compatible with augmentative releases of the aphid parasitoid *Aphidius colemani* Viereck (Hymenoptera: Braconidae) when host aphids were present on the crop [96]. Panels of such netting have also been used in conjunction with ultraviolet lights and pheromone lures as an ‘attract-and-kill’ strategy on perimeter rows of pear orchards to protect developing fruit from brown marmorated stink bugs as the adults migrate in from surrounding hedgerows and field crops [97]. Such a strategy—which would involve limited materials and a one-time effort to set up and take down—might represent a considerable saving in time, effort and money for small-scale growers dealing with this and other ‘perimeter’ pests.

##### Fruit Bagging

For some crops, an alternative to netting entire rows or blocks is to enclose individual fruits with paper-, micro-perforated polyethylene-, or fine mesh bags to prevent pre-harvest pest damage. In Japan and China, fruit bagging is an established practice for optimizing the quality and finish of various tree- and vine fruits, including apples, pears, peaches, loquats and grapes. Fruit bagging has been used elsewhere to reduce pest damage in mangoes, guava, litchi, pomegranates, citrus fruits and pitaya (dragon fruit) [98,99,100,101,102].

Small-scale growers of tree fruit crops may lack the specialized equipment necessary for spraying tall trees, and/or lack access to appropriate insecticides (or adjuvants) in pack sizes suitable for their needs. It can therefore be difficult for them to achieve successful chemical control of internal pests of tree fruit, particularly species that undergo multiple generations that must be treated more than once per generation. Hence, a single, well-timed application of fruit bags, while impractical on a large scale, can be useful for small-scale commercial growers anticipating high returns (e.g., for organic fruit), or for home gardeners [103,104]. In Asia, where fruit bagging is more widely practiced, several hand-held application devices are available to increase bagging efficiency, with various improvements currently under development [99,105].

In the US, fruit bagging has been shown to significantly reduce damage by direct pests of apples and peaches, including codling moth, peach twig borer (*Anarsia lineatella* Zeller) (Lepidoptera, Gelechiidae), stink bugs (including brown marmorated stink bug), and birds [103,106,107]. However, some materials work better than others under different circumstances, and, in some cases, also work better on some cultivars than others. There is evidence that the color of the bagging material may affect the results: in apples, for example, Sharma et al. [108] found that fruit bags of light-yellow material were significantly more effective at reducing San Jose scale (*Comstockaspis perniciosus* (Comstock)) (Hemiptera: Diaspididae), apple scab (*Venturia inaequalis* (Cooke) G. Winter)), and the fungal complex associated with sooty blotch/flyspeck, than were blue, green, or red bags.

Relatively little research has been conducted on the use of bagging for pest management in fruiting vegetables. Nevertheless, where high value vegetables (such as organic tomatoes (*Solanum lycopersicum* L.) (Solanaceae)) are subject to attack by internal pests (e.g., larval Lepidoptera such as *Helicoverpa zea* (Boddie) (Noctuidae) or *Tuta absoluta* (Meyrick) (Gelechiidae)), fruit bagging can be both effective and lucrative, particularly with bags that can be re-used for more than one season [109,110]. The practice of bagging pepper fruits is also reported to be increasing in parts of Asia, in part to reduce both pest damage and pesticide residues on the fruits [111].

In summary, exclusion techniques can be cost-effective and successful components of IPM for small-scale producers, provided that certain precautions are taken during their development and implementation. Critical considerations during the research phase include evaluating possible impacts on crop microclimate (with associated effects on pests and diseases), as well as effects on crop yield and quality [74,75,76]. For some crops, supplemental pollination may be required if pollinators are excluded during the flowering period [78,112].

Finally, while exclusion methods are popularly viewed as less damaging to the environment than chemically-intensive pest management programs, most nets are at present manufactured from non-biodegradable plastics using fossil fuel feedstocks, prompting concerns over the environmental impact of their manufacture and ultimate disposal. A recent review addresses the possibility of overcoming these drawbacks by fabricating exclusion materials from biopolymers (made from biomass feedstocks), which may have a similar life expectancy to current nets, with the added benefits of being generally carbon-neutral and often biodegradable [113].

#### 4.3.2. Mating Disruption and Mass Trapping

Pheromone-based mating disruption is generally considered appropriate only for relatively large-scale, uniform plantings (approx. 2–4 ha, depending on product and target species). However, for pests with very limited dispersal behavior, mating disruption can be an effective way of reducing either initial colonization or established populations even in small plantings (less than 0.5 ha in size).

For example, in a five-year study of the effect of pheromone-based mating disruption on the peachtree borer (*S.* *exitiosa*), infestation levels in a 0.08 ha peach orchard declined from 57.5% to 8.4% after two years of pheromone treatment, and slowly increased (to 16.9%) over the following three years when pheromone treatments were discontinued [37]. Similar results were obtained for the leopard moth *Zeuzera pyrina* (L.) (Lepidoptera: Cossidae) when mating disruption products were applied for two years in small (0.24 ha) olive blocks [114]. In both of these studies, the target pest was already established at the test sites, and the adult females of both species have been reported to show very limited dispersal from their natal trees.

Pheromone-based mating disruption may also have potential for managing non-Lepidopteran pests with poor dispersal abilities. These may include, for example, the swede midge (*C. nasturtii*) for which mating disruption was shown to reduce damage by 59% in small-scale trials. For this species, however, synthesis of the pheromone is currently considered too expensive for widespread use in mating disruption [115]; in the future, however, it may be possible to use it in a more cost-effective way as part of a mass-trapping system, as has been proposed for other ceccidomyid midges [116,117].

For horticultural pests, mass trapping is another technique that may be more cost-effective for small-scale growers than for those with larger acreages, provided that systems can be developed that capture sufficient numbers of the target pest(s) before they can reproduce or damage the crop [118]. For example, an experimental mass-trapping technique developed for managing Japanese beetle (*Popillia japonica* Newman) (Coleoptera: Scarabaeidae) on small-scale fruit farms captured very high numbers of beetles (more than 10 million over three years) and kept crop damage low [119]. In a novel extension of this strategy, the collected beetles were subsequently composted and converted into high-quality soil amendments that could help offset the costs of the traps and lures [120].

#### 4.3.3. Sanitation

For some pests, sanitation methods that may be too labor-intensive on a large scale can be more manageable in smaller plantings. Such approaches include, for example, shortening the harvest interval for management of spotted wing drosophila in berry crops [121,122], prompt harvesting and removal of over-ripe fruit to minimize damage to berries and stone fruit by the green fig beetle *Cotinis mutabilis* (Gory and Percheron) (Coleoptera: Scarabaeidae), and winter removal/destruction of galls of the blueberry stem gall wasp (*Hemadas nubilipennis* Ashmead) ((Hymenoptera: Pteromalidae) prior to spring emergence of the adults. In some cases, appropriate sanitation alone can provide sufficient control (L. Candelaria, personal communication), while in others it must be combined with other tactics as part of an overall IPM strategy [121].

### 4.4. Opportunities for on-Farm Education and Participatory Research

In 2018, 55% of the global population was estimated to live in urban centers, with the most urbanized regions being Northern America (82% living in urban areas), Latin America and the Caribbean (81%), Europe (74%) and Oceania (68%) [123]. As opportunities for these urban populations to experience direct contact with the land have diminished, there has been a corresponding increase in interest in farms as learning environments [124] not only for youth, but for adults as well. Educational farm walks and tours have long been popular as Extension methods, and there is considerable scope for individual farmers to share their knowledge in this way, and perhaps to engage the wider community in collaborative *in situ* IPM trials. There is evidence that active involvement in such trials helps support the adoption of IPM by farmers and gardeners by reducing their use of broad-spectrum insecticides and increasing their incorporation of biological control [125,126,127]. While such activities usually require some input from professional IPM practitioners to support the participants, a linked network of such projects could leverage their involvement and extend their impact while minimizing costs.

## 5. Conclusions

This review has highlighted some of the principal characteristics of small-scale farms (and farmers) in developed economies, and examined how some of these factors have contributed to challenges faced by this sector in relation to IPM adoption and implementation. While small-scale farmers undoubtedly face many and varied challenges (including land tenure/availability, access to markets, etc.), from the point of view of pest management, there is reason to be cautiously optimistic. In addition to crop protection methods that are applicable to virtually any scale of production, as discussed above, there are a variety of IPM tactics that may be easier to implement (or more cost-effective) on small-scale farms compared to larger ones. Moreover, new approaches to pest management are constantly under development, and some will undoubtedly be applicable to small-scale cropping systems. In this regard, methods of modifying crop production to manipulate beneficial soil microorganisms that can increase plant health and pest resistance may be particularly promising [128,129,130].

Furthermore, while good farmers—of all scales—are inherently observant, knowledgeable and innovative, small-scale growers have the advantage of a very intimate daily association with their crops that is not possible at a larger scale. This in turn gives them more opportunities to make the close observations that can provide critical insights into pest problems and which ultimately may help them develop possible solutions suited to their own particular constraints. In this respect, the lack of relevant educational resources and support from agricultural research establishments may not be the impediments they may appear to be at first. Before its current rise to prominence, organic farming was in much the same situation, and it can be argued that such conditions may—by necessity—foster more diverse and more innovative approaches to problem-solving than may arise from traditional ‘top-down’ approaches.

Nevertheless, a degree of collective organization—for example, in the form of grower associations or co-operatives—could be beneficial in representing the needs of small-scale growers at the research or policy levels, or for the joint purchasing of inputs or equipment. In this way, challenges as disparate as a lack of political influence and the limited availability of scale-appropriate inputs could be addressed through concerted action. Furthermore, growers collaborating in this way could share the costs of appropriate agricultural innovations that may initially be too costly for individuals. An example might be the recently developed autonomous robotic weeding machines that can weed up to 1000 row-meters per hour [131]; at this work rate, a group of perhaps 5–10 small-scale growers could jointly purchase and share a single machine and still be able to stay ahead of developing weed pressures on each farm. 

Finally, in addressing the subject of IPM on small-scale farms, the overall importance of such farms should be considered in their wider context. Even in developed economies, small farms are important not only for food production, but also in relation to other biological and societal benefits, such as their contribution to local economies, their role in conserving biodiversity and rural landscapes, and in maintaining various other aspects of cultural and culinary heritage (including dietary diversity) [12]. In the future, with predicted increases in catastrophic weather events and probable increasing pressure on food distribution systems, small diversified farms will also become increasingly important in maintaining local food security and community resilience. With that in mind, it behooves policy makers, researchers, and IPM practitioners alike to increase their efforts at overcoming some of the challenges presented here, and embrace instead the many opportunities offered by such systems.
